# Large-scale additive manufacturing with bioinspired cellulosic materials

**DOI:** 10.1038/s41598-018-26985-2

**Published:** 2018-06-05

**Authors:** Naresh D. Sanandiya, Yadunund Vijay, Marina Dimopoulou, Stylianos Dritsas, Javier G. Fernandez

**Affiliations:** Singapore University of Technology & Design, 8 Somapah Road, 487372 Singapore, Singapore

## Abstract

Cellulose is the most abundant and broadly distributed organic compound and industrial by-product on Earth. However, despite decades of extensive research, the bottom-up use of cellulose to fabricate 3D objects is still plagued with problems that restrict its practical applications: derivatives with vast polluting effects, use in combination with plastics, lack of scalability and high production cost. Here we demonstrate the general use of cellulose to manufacture large 3D objects. Our approach diverges from the common association of cellulose with green plants and it is inspired by the wall of the fungus-like oomycetes, which is reproduced introducing small amounts of chitin between cellulose fibers. The resulting fungal-like adhesive material(s) (FLAM) are strong, lightweight and inexpensive, and can be molded or processed using woodworking techniques. We believe this first large-scale additive manufacture with ubiquitous biological polymers will be the catalyst for the transition to environmentally benign and circular manufacturing models.

## Introduction

Cellulose and chitin are the first and second most abundant polymers on the surface of the Earth^1^, and consequently a recurrent topic of research for their potential utilization in manufacture^2,3^. Typically, cellulose is associated with plants and chitin with arthropods, however the natural occurrence of both biopolymers as structural components broadens to most kingdoms of eukaryota and bacteria^1^. Despite their abundance, they rarely coappear in the same organism. One exception of this are certain species of oomycetes^4^, a large class of eukaryotic organisms. Oomycetes grow in a mycelial form as fungi. However, in contrast to fungi which are characterized by a chitinous wall, oomycetes’ cell walls, and those of their close relative hyphochytrids, are predominately based on cellulose^4,5^.

In the last few years, the pathogenic nature of some oomycetes has motivated a meticulous characterization of their wall singularities, as possible targets for disease control^6^. Those studies have also shed some light on the characteristics of natural structures made of chitin and cellulose. This knowledge has direct application on the development of bioinspired materials. We now know oomycetes are not a homogeneous population but a combination of members with at least three distinctive cell wall types^7^. While those walls are mostly composed of cellulose, they also contain low concentrations of chitinous polymers, comprising up to a 10% of the cellulose content^8^. Inspired by this idea we studied the effects on manufacturability of cellulose by the addition of small amounts (<15%) of highly deacetylated chitin (i.e. chitosan) and the influence of the chitinous polymer in the ability of the composite to form three-dimensional structures.

The objective of our research is to apply the principles of the cell wall of fungi and oomycetes to produce a general manufacture system based on three premises: (i) The resulting bioinspired composite must be made by its natural constituents; (ii) Components must be available and abundant in every habitat on earth; (iii) Cost, environmental impact, and scalability must enable generalized use. Due to recent research on the oomycetal walls we know that chitin and cellulose produce structural composites in their natural form^6^, without being regenerated, and therefore our criteria (i) and (ii) are theoretically possible. Additionally, both molecules are common industrial byproducts with a combined cost in the range of commodity plastic^9–12^, being exceptional, and probably unique, biological candidates to fulfill criterion (iii).

Our research focuses on the reproduction of the synergies between molecules in biological composites, and we approach this by artificially associating structural biomolecules in their organization in living systems^13^. This differs from the two predominant approaches to bioinspired materials, based on the reproduction of natural composites with synthetic materials of known manufacturability, and from transforming natural components to fit in already existing manufacture techniques^14^. The later, has given rise to cellulose modified to form thermoplastic polymers (celluloid), and regenerated to form films (cellophane) or fibers (rayon). These chemical transformations and dissolutions require strong organic solvents and hazardous pollutants such as acetone, carbon disulfide, and sulfuric acid^15^. As a result, while some of those variants of cellulose were very popular in the 1970′s, their current use has declined to small niche markets^16^.

**Figure 1:**
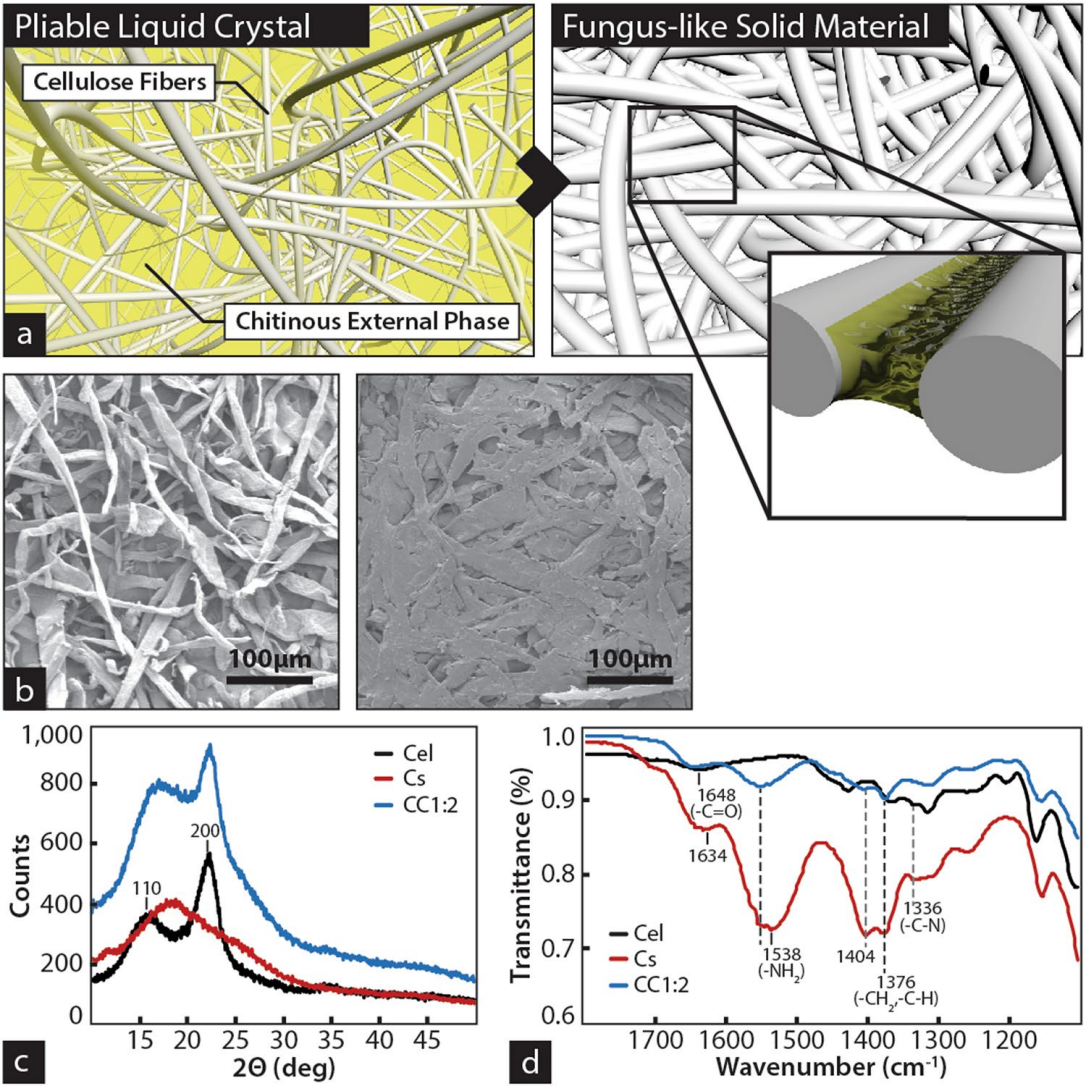
Supramolecular organization of fungus-like mimetic materials. (**a**) Cellulose fibers from plant origin are dispersed in chitosan solution. After removal of the water, chitosan crystallize in between fibers. In the process the sterically available amino groups in chitosan not involved in the crystal conformation react with the free hydroxyl groups on the surface of the cellulose fiber. As the chitosan losses intermolecular water, the polymer crystal reduced volume brings together the cellulosic fibers into a solid composite. (**b**) Scanning electron microscopic images of the cellulose fibers (left) and their structure in the chitinous composite (12.5% chitosan concentration). (**c**) X-ray diffraction pattern of the composite and their constituents. The data shows a cellulose I polymorph unaltered during the formation of the composite. A relaxation on the crystal structure, reflected in a shift of the 002 reflection, could be caused for the hydrogen bond of the cellulosic hydroxyl groups with chitosan, reducing the amount of cellulose-cellulose intermolecular hydrogen bonds. (**d**) FTIR fingerprint of the Chitosan-cellulose composite. The amino groups of the chitosan shifted from 1538 to 1556 cm^−1^ and the band associated with the hydroxyl groups of the cellulose shifted from 1640 to 1648 cm^−1^ indicated the interaction between amino groups of chitosan and hydroxyl groups of the cellulose.

In contrast with the chemical stability of cellulose, chitin with low degree of acetylation (i.e. chitosan) contains enough protonatable groups to enable its dispersion in low concentrations (i.e. 1% v/v) of acetic acid^17^ (e.g. table vinegar 4–10%). Chitosan in solution is driven into a liquid crystal by partial removal of the intermolecular water^18^ and in that state we used it as external phase to form colloidal dispersions of cellulose fibers. Further removal of the water molecules results in full crystallization of the chitinous polymer and formation of a solid chitosan-cellulose composite (Fig. 1a). Scanning Electron images reveals the original microscopic fibrous structure of the cellulose in the composite (Fig. 1b), while X-Ray diffraction confirms that the cellulose I crystal conformation^19^ is intact (Fig. 1c). The FTIR spectra of the chitosan and cellulose composites reveals the predominant interaction between the amino and hydroxyl groups respectively^3,20^ (Fig. 1d). Separate phases of the two components are observed at large concentration of chitosan, turning into a homogenous composite, without phase separation, but with a single decomposition temperature when the concentration of chitosan is under 30% of the total weight (Supplementary Fig. 1).

**Figure 2:**
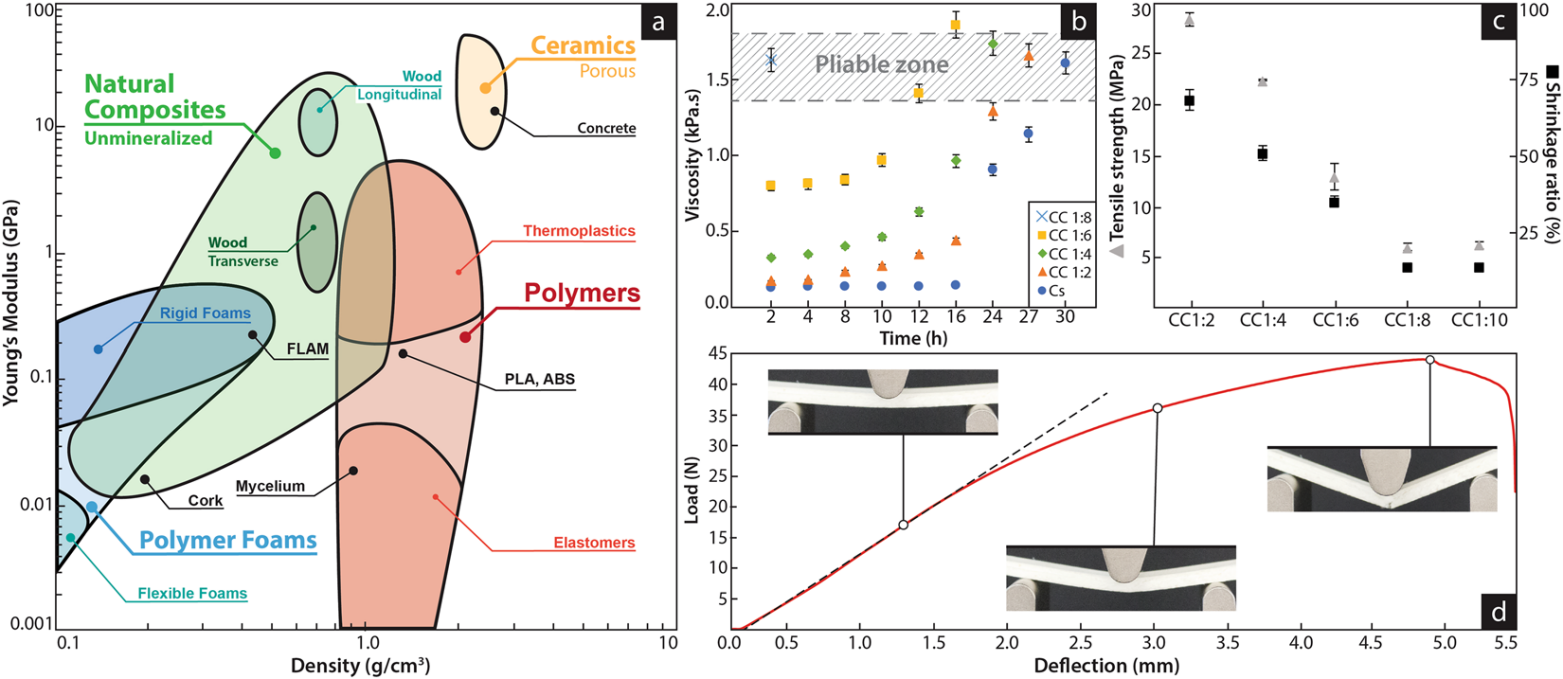
Mechanical characteristics of fungus-like biomimetic materials. (**a**) Ashby plot showing the distribution of density and stiffness of natural and synthetic material commonly used in manufacture. Those relevant to this study have been highlighted, while the specific function of the fungus-like bioinspired material reported is labeled as “FLAM”. (**b**) Viscosity in terms of time for composites of variable amounts of chitosan. The highlighted area represents the range of viscosities suitable for manufacturing techniques, where the material can be extruded, and conform and retain a shape. (**c**) Determination of the optimal concentration regarding the balance between mechanical properties (tensile strength) and manufacturability (shrinking). (**d**) 3-point fracture test. Similar to other natural composites, the resulting material is designed for multifunctional structures, balancing strength and stiffness. The 100 × 16 × 6 mm and 3 g FLAM sample shows ductile characteristics; it holds 2.55 kg elastically, after that the internal structure starts to deform, resulting in failure when the load reaches 4.37 kg (Supplementary Movie 1).

We further explored the manufacturability of the chitin-cellulose fungus-like material by evaluating the effect of the chitin concentration on the ability of the composite to reach a pliable state and retain shape. Chitosan is introduced in the composite as a water solution. As a result, composites with large amounts of chitosan (>12%) require removal of part of that water until the material reaches a state able to conform and retain a three-dimensional shape (Fig. 2b). After solidification, composites with low amounts of chitosan (<8%) show slender mechanical properties, attributable to its inability to fully bind the cellulose fibers. In contrast, high amounts of chitosan (>17%) produce strong internal forces as the polymers loses water and shrinks, undermining the integrity of structure (Fig. 2c). An inherent optimal ratio of 1:8(w/w) chitosan:cellulose, produces a pliable composite, where no removal of water is required, with minimum shrinking and an unexpected low water uptake (Supplementary Fig. 2). Interestingly, recent studies on the oomycete wall draw similar conclusions for the ratios of chitin/cellulose^7^, and also reported an abnormal water uptake on the cell wall when chitin production is disrupted^6^. The fungus-like additive material (FLAM) has a cost of about 2 $/kg and, with a young modulus of 0.26 Gpa and a density of 0.37 g/cm^3^. It is wort noting that while the cost of FLAM is in the range of commodity plastics, is more than ten times lower than those of common filaments for 3D printing, such as PLA and ABS with an average cost of 20–30 $/kg. The mechanical characteristics of FLAM are within the range of natural cellulosic composites such as medium to low density woods and high-density foams, commonly used in product design, construction, aviation and automotive industries (Fig. 2a,d, Supplementary Fig. 3, Supplementary Table 2, and Supplementary Movie 1). Nevertheless, they are significantly different to any characterized natural material. FLAM is a reproduction of a natural material synthesized at the microscale (i.e. the oomycete wall), so it is probable its characteristics are similar to uncharacterized materials existing only at that scale. The only reported material with almost identical mechanical characteristics to FLAM is high-ending rigid polyurethane foam (i.e. pcf-30), commonly used for production of synthetic bones^21^.

**Figure 3:**
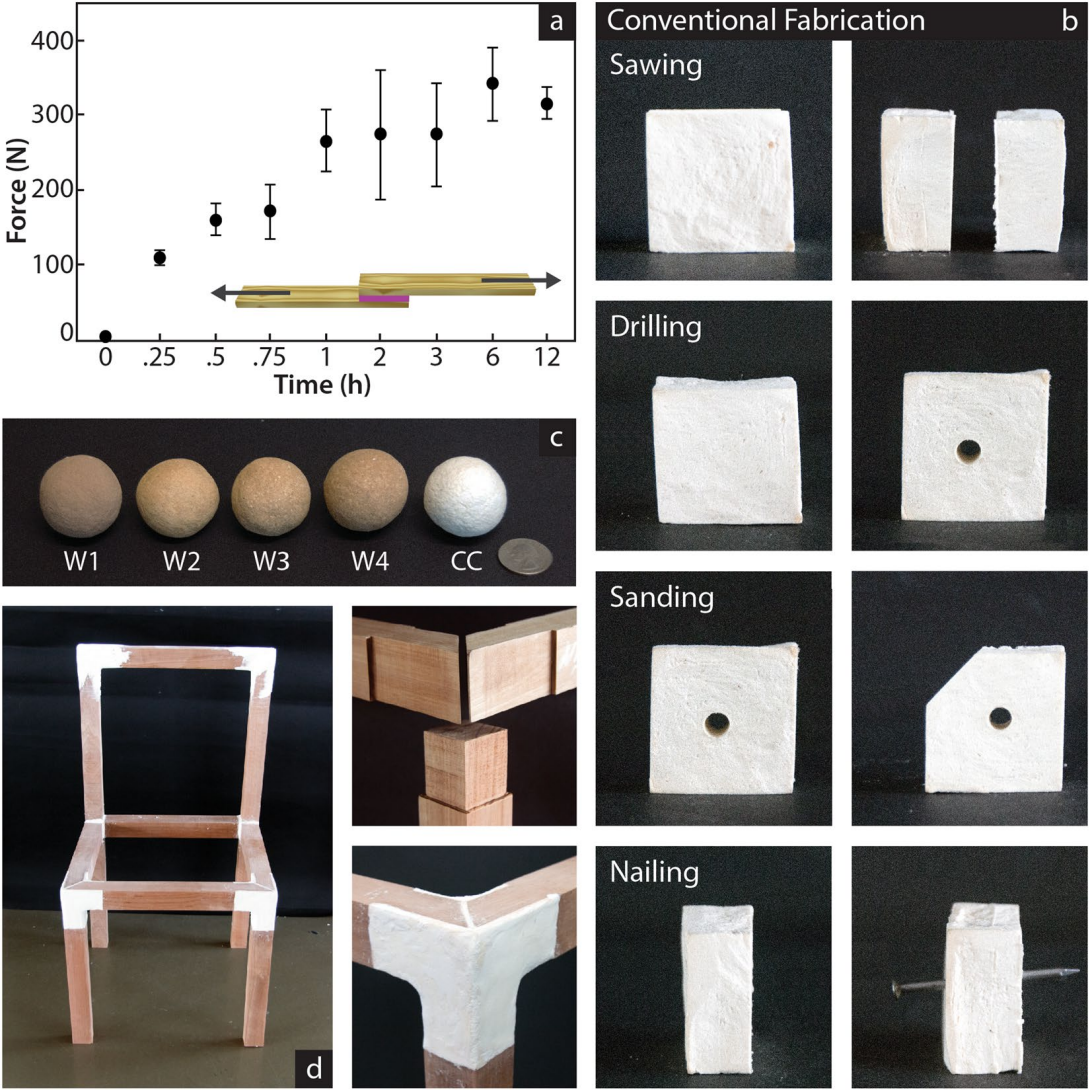
Additive properties of fungus-like materials and their use in woodworking techniques. **(a**) Adhesion force with respect time following the standard test of adhesion (ASTM D5868) with respect to time. Full strength is achieved after 1 hour, from that point 21 mg of dry FLAM covering an area of 9.3 × 9.3 mm holds the equivalent to 29.02 ± 6.35 kg. This ability of the material to attach to cellulosic composites (included itself) enables its use in additive manufacturing. (**b**) Use of FLAM in conventional woodworking techniques. A 4 × 4 × 4 cm FLAM cube is sawn in two halves, one of the halves is then drilled and then sanded down to remove one of the corners. A nail is hammered through the other half. (Supplementary Movie 2) (**c**) Composites made with different sources of cellulosic materials. Samples one to four (from left) are made of wood byproducts of different qualities and sources, while the right sphere (labeled “CC”) is made of pure cellulose (Supplementary Tables 1 and 2). (**d**) Use of the material in combination with pieces of solid wood to produce a functional chair. All pieces are attached uniquely by the FLAM material. The fungus-like biomimetic material can be casted, 3D printed, molded but also modified using regular woodworking techniques (Supplementary Fig. 4).

While most cellulosic and chitinous natural structures also include other organic and inorganic components, the interaction between cellulose and chitosan has proven sufficient to form solid composites. These interactions are strong enough even in presence of disruptive components, such as lignin or hemicellulose, enabling the formation of wood flour-based composites (Fig. 3a). The mechanical characteristics of these wood-based composites are significantly lower than FLAM based on pure cellulose (Supplementary Table 2), yet they enable a generalization of the technology to many other sources of unprocessed byproducts. In the US for example, 14% of the municipal waste is wood^22^, while industries such as agriculture, food, textile, and paper, produce high amount of waste with high cellulose content. We tested wood flour from three different sources and purities, producing composites and objects with small mechanical differences with respect to the waste source or the manufacture method (Fig. 3b, Supplementary Fig. 3, and Supplementary Table 2). This chitinous property of binding cellulosic composites is retained by FLAM as when FLAM is deposited on wood it naturally forms adhesion bonds able to hold an average of 33.5 ± 7.3 kg per cm^2^ (Fig. 3a). This feature broadens the applicability of the material to an unprecedented range of strategies: it can be machined using common woodworking techniques such as sawing and sanding, (Fig. 3c and Supplementary Movie 2) as well as used in combination with hardwood and other cellulosic components (Fig. 3c, Supplementary Fig. 4, and Supplementary Movie 3). More interestingly, the property of FLAM to bond with cellulosic composites also include fusing with itself, enabling its use in additive manufacturing (Supplementary Fig. 3).

**Figure 4:**
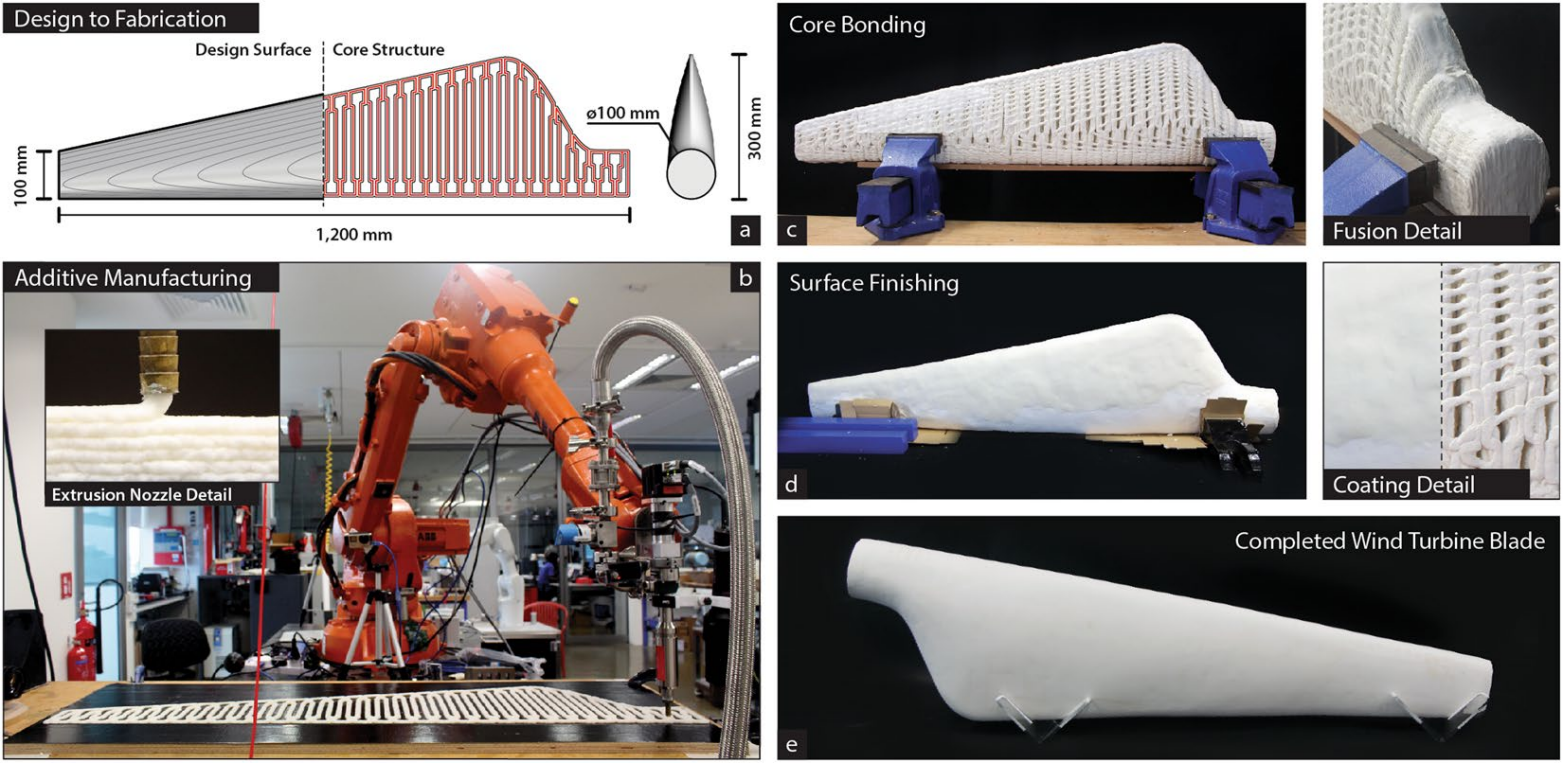
Additive manufacture of fungus-like materials. (**a**) Design of the wind turbine blade fully made of FLAM. The inner core of the blade, built by additive manufacturing, is designed to allow ventilation and reduce weight. The outer shell is produced by coating the core with a 3 mm layer of FLAM. After drying the outer shell is sanded down to produce a smooth surface. (**b**) FLAM material is dispensed through a 7 mm diameter nozzle to form beads and then layers of material. The pressure is controlled by a closed loop system between a high-pressure tank (1.2 MPa) supplying material and an auger screw before the nozzle. Hot air is focused on the extruded layer just after deposition to accelerate water evaporation. The printing head is mounted on a 20 kg payload six-axis industrial robotic arm with a stationary horizontal reach radius of 1.65 m (Supplementary Fig. 6). (**c**) FLAM printing of the layers to support the structure of the wind turbine blade. The blade’s core was printed in two halves, each taking about 1 h and 24 h to dry. The average printing speed is of about 50 mm/s and 2.8 cm^3^ of FLAM per second (Supplementary Movie 3). Two halves of the inner core are assembled together using FLAM as adhesive agent. (**d**) The blade is coated by a layer of FLAM manually spread over the inner core. Because the ability of the material to be post processed, imperfections can be removed in post-processing stages. (**e**) After the outer skin is dried, it is sanded down in two steps of decreasing grit. The 1.2 m blade is estimated to be 50% hollow inside and has a weight of 5.28 kg. (Supplementary Movie 4 and Supplementary Fig. 5).

Because of its abundance and biodegradability, the use of cellulose in a versatile manufacture approach such as additive manufacturing has broad technological and economic implications^23^. In spite of extensive past and current research to adapt cellulose to 3D printing, progress is still riddled with obstacles such as use of hazardous solvents^16,24,25^, lyophilizing small cellulosic scaffolds^25^ and contamination by mixing the polymer with commodity plastics^26,27^. We developed a large scale 3D printing system specific for FLAM natural materials based on the Direct Ink Writing (DIW) principles^28^ (Fig. 4b, Supplementary Figs 3, and 6). Core components include a precision robotic dispensing system and associated design-to-manufacture software tailored for FLAM. To demonstrate the capabilities of the system, we 3D printed a 1.2 m long wind turbine blade (Fig. 4a). The blade is comprised on two symmetric inner core parts (Fig. 4c) hollowed to accelerate evaporating hardening and fused together using FLAM. The tribological function of a turbine blade makes it incompatible with the stepped surface produced by additive manufacturing^29^. To improve surface finish and potential aerodynamic performance, the blade was coated by a thin layer of FLAM and subsequently polished (Fig. 4d). Additional coatings with other materials can be used to add functionality to the surface, such as self-cleaning, reduced air resistance, or waterproofing. FLAM has a density less than one half that of the lightest commercially available 3D printed filament (Polyamide, 0.95 g/cm^3^), resulting in a lightweight finished blade of 5.28 kg (Fig. 4e, Supplementary Fig. 5, and Supplementary Movie 4).

No technology has ever been reported to possess the unique characteristics of FLAM: made of the two most abundant and broadly distributed components on earth, lightweight, cost in the range of commodity plastics, suitable for large-scale manufacture, lack of harmful solvents/pollutants, compatible with cellulosic composites, completely biodegradable out of composting systems. Additionally, to our knowledge, there is no other biotic material with the adaptability to be casted, molded, sanded, sawn and 3D printed. It is expected that FLAM can delocalize general manufacture and meet the emerging needs of sustainable manufacture, large-scale fabrication, and circular economy, as well as be a disruptive technology across multiple industries, including the architectural, aerospace, and biomedical, enabling the development of many more areas and new manufacture strategies beyond the current reach of technology.

## Materials and Methods

### Materials

High Purity cellulose (JELUCEL® HM400X; fiber size <200 μm) and wood flour- W1 (JELUXYL® WEHO 500; grain size <500 μm) were supplied by JELU-WERK GmbH & Co. KG, Germany. Sawdust- byproducts of the woodwork machines was collected from fabrication lab-SUTD (W2), Singapore. Chitosan used in the manufacturing process was purchased from i-CHESS chemicals Pvt Ltd., India (Table S1). Chitosan (medium molecular weight; 75–85% deacetylated) used for molecular characterization and glacial acetic acid, were purchased from Sigma-Aldrich, Singapore. All chemicals were used as received. FLAM preparation: Chitosan solution (3% w/w) was prepared by dissolving chitosan in 1%(v/v) acetic acid. Fungus-like additive materials (FLAM) were prepared by mixing chitosan solution and cellulose (C:C) in varying ratio (dry w/w) 1:1, 1:2, 1:4, 1:6, 1:8 and 1:10, and were used to cast films for molecular characterization, specimens for mechanical characterization and hand-casted structures followed by oven drying at 50 °C. The FLAM to manufacture macrostructures were prepared in a 1:8 ratio (C:C) by mixing in an Industrial dough mixer (B25-25L, Yiwu Sneeceman, China) and 90 rpm.

### Molecular Characterization and imaging

Scanning electron microscopy: The surface morphology of FLAM films was examined using a Field Emission Scanning Electron Microscope (FESEM, JEOL, JSM-7600F) at 5 kV accelerating voltage. Samples were mounted onto a carbon tape on an aluminum stub and sputter coated with gold for 30 s. X-ray diffractionmetry: X-ray diffraction diagrams of cellulose and composite films were obtained on a X-ray Diffraction System (XRD)- D8 Discover Bruker, using nickel-filtered CuKα radiation (λ = 0.15418 nm) operated at 40 mA and 40 kV, with a scan speed of 3°/min having set the 2θ angle 2° to 60°. FTIR: FTIR spectra of composite films were obtained using a Fourier transform infrared (FTIR) spectrometer, VERTEX 70 FTIR (Bruker optik GmbH), with a resolution of 4 cm^−1^ and accumulation of 32 scans between 4000 to 400 cm^−1^ on ATR mode. TGA: Thermogravimetric analysis (TGA) of samples were carried out on a Thermogravimetric Analyzer TA Q50, TA instruments, using a temperature ramp from 30 °C to 700 °C at a heating rate of 10 °C/min in a nitrogen atmosphere. Rheological measurements: The flow behaviors of FLAMs were determined by measuring the dynamic shear viscosity with different shear rates (0.05–30 s^−1^) on a Rheometer (HR-2 Discovery Hybrid Rheometer, TA instruments equipped with Environmental Test Chamber), using parallel plate geometry (40 mm diameter; 500 μm gap). FLAMs with ratio 1:2, 1:4 and 1:6 were kept in an oven at 50 °C with frequent stirring and viscosity of each sample was measured periodically at 0, 2, 4, 6, 8, 10, 12, 16, 20, 24, 27 and 30 h, until viscosity reached to the pliable range. Prepared pliable composites were further used for tensile strength and shrinking behavior measurements.

### Mechanical testing

Tensile strength: FLAMs with optimized pliable viscosities were cast in a dog-bone shape mold with 100 × 16 × 6 mm size in the reduced section. FLAM (CC 1:8) was printed on manufacturing system (Figure S6) of 150 × 12 × 4 mm size (single filaments), 150 × 18 × 4 mm (double filaments) and 150 × 18 × 8 mm (quadruple filaments) size to prepare specimen to evaluate tensile strengths of the printed parts (Figure S3). The cast and printed test specimens were allowed to dry in an oven at 50 °C for 24 h and were subjected to a tensile test adapted from ASTM standards D1037-12, using a UTM (Universal Testing Machine- Instron 5943 and Instron 5942) equipped with 1 kN and 100 kN load cell with a cross head speed of 4 mm min^−1^ at ambient conditions. 3-point bending test: FLAM (CC 1:8) was cast in a mold with 100 × 16 × 6 mm internal size and was also printed of 150 × 12 × 4 mm size (single filaments), 150 × 18 × 4 mm (double filaments) and 150 × 18 × 8 mm (quadruple filaments) size followed by drying in an oven at 50 °C for 24 h. The test specimens were tested for the flexural test following ASTM standards (D1037–12) on UTM equipped with 3-point flexure test fixtures. A support span of 60 mm and head speed of 4 mm min^−1^ was used. Compressive strength: FLAM (CC 1:8) was casted in a 50 × 50 × 50 mm acrylic mold and was also printed of same size on manufacturing system followed by drying in an oven at 50 °C. The printed cube was sanded with 240 grit before the test was performed. Compression test was performed on UTM equipped with parallel compression fixtures with a cross head speed of 4 mm min^−1^. Bulk adhesive strength: Adhesive performance of FLAM (CC 1:8) was analyzed by single-lap shear test in accordance with the ASTM standards D5868−01(2014). Basswood panel of 3 mm thickness was cut into 9.8 mm × 100 mm size with a lesser cutter to prepare adherends. Wet FLAM (*ca*. 100 mg wet; ~21 mg dry) was applied on the surface of adherends covering 9.8 mm × 9.8 mm area and another adherend was placed on the top clamping it by bulldog clip. Samples were placed in a heating oven at 50 °C to cure and lap-shear was tested to failure on UTM using 1 kN load cell with a cross-head speed of 10 mm min^−1^, at ambient condition. Statistical analysis: Experimental data were expressed as the mean ± standard deviation, with at least triplicate measurements.

### Moisture absorption measurement

Moisture absorption measurement was carried on completely dried and pre-weighed mold-casted cuboids with the size 30 × 15 × 15 mm. The samples were placed in the humidity chamber at 95% humidity at 25 °C for certain time.

The moisture absorption (%) was calculated using the following equation (1)

1 $${Moisture}\,{absorption}\,( \% )=\frac{{{M}}_{{w}}-{{M}}_{{d}}}{{{M}}_{{d}}}\times 100$$

Here, M_d_ is the weight of the dry specimen before placing in humidity chamber and M_w_ is the weight after moisture absorption at certain time.

### Additive Manufacturing System

Positioning system: The 3D printing system is comprised of a commercial six-axis articulated industrial robot with maximum horizontal reach of 1.65 m and 20 Kg payload at the flange mounted on a purpose design and built hydraulic scissor-lift mobile platform for extending its work envelope horizontally indefinitely and up to 3.7 m vertically (Figure S6). Material Transport System: The FLAM extrusion system is comprised of a commercial stationary 15 L bulk unloading pump system and a precision dispensing unit 1.7 ml/rev at maximum 125 RPM mounted on the robot’s flange driven by smart servo motor. Both units are rated for highly viscous materials up to 20 bar supply pressure, use anti-sheer and anti-pulsation auger screw cavity pump design. Integration Components: Signal interfacing and communication between the robot’s controller and extrusion system uses bespoke Programmable Control Logic software and hardware. Miscellaneous electrical and mechanical interfaces between robot and extrusion system such as mounting plates, nozzles and couplings were fabricated at SUTD FabLab. Design and Control Software: Parametric design software was developed for the design of prototypes and blade artifact and translation of design geometry to machine instructions for off-line robot programming. Material compaction vertically and expansion horizontally were encoded to create a pre-set model from design geometry. Subsequently, machine pathing algorithms were created for interior core structure that account for per layer path continuity to reduce start/stopping the dispenser and eliminate artifacts such as tailing, material feed and speed calibration for maximizing production time without inducing bead deformation by dragging, overhang rate to reduce sagging, cross bead overlap for improving horizontal fusion, interior cavity area increase for accelerating evaporative hardening. Process Parameters: The nozzle internal diameter used is 7 mm and layer height 3 mm for interlayer adhesion which results into a capsule shaped bead profile with approximately 13 mm width. The flow rate, approximately 2.8 ml/sec with feed rate of 50 mm/sec, were derived from design experiment model.

## Electronic supplementary material

Supporting Online Material

Movie 1 - 3-points fracture test

Movie 2 - Examples of woodworking techniques on FLAM

Movie 3 - 10min printing (cylinder)

Movie 4 - Fabrication of a FLAM windmill blade by additive manufacturing
