# Supplementary material for: Large-scale additive manufacturing with bioinspired cellulosic materials
Source: Sci Rep. 2018 Jun 5;8:8642. doi: 10.1038/s41598-018-26985-2 (PMC5988822; doi:10.1038/s41598-018-26985-2)
Supplement: Supplementary file 1 — Supporting Online Material [file 41598_2018_26985_MOESM1_ESM.pdf]

Supporting Online Material

Large-scale additive manufacturing with bioinspired cellulosic materials

Naresh D. Sanandiya, Yadunund Vijay, Marina Dimopoulou, Stylianos Dritsas & Javier G. Fernandez*

*To whom correspondence should be addressed. E-mail:

Javier.fernandez@sutd.edu.sg

This material includes:

Table 1 Physical characteristics of raw materials
Table 2 Mechanical characteristics of FLAMs using wood byproducts from different sources

Figure 1 Thermogravimetric analysis (TGA) and derivative thermogravimetric (DTG) curves of chitosan/cellulose composite with different ratio.
Figure 2 Moisture absorption study of FLAMs
Figure 3 Mechanical characterization of 3D printed FLAM
Figure 4 Development prototypes created using conventional, additive manufacturing and combinations thereof methods.
Figure 5 Sequence of turbine blade production combining digital and analog fabrication techniques to demonstrate the versatility of FLAM materials
Figure 6 Large-scale 3D printing setup.

Movie 1 3-points fracture test
Movie 2 Examples of woodworking techniques on FLAM
Movie 3 10min printing (cylinder)
Movie 4 Fabrication of a FLAM windmill blade by additive manufacturing

Table 1: Physical characteristics of raw materials

	Chitosan	*Cellulose [HM 400X]*	*Wood Flour* W1 *[WEHO 500]*	Wood Flour W2
Bulk Density[g/cm3]	0.2	0.13	0.18	0.25
Appearance	Yellowish powder	White powder	Yellowish powder	Brown powder
Particle size(μm)	100-4000	~32	75-180	100-600
Source	Shrimp shell	softwood	Spruce	Plywood dust

Table 2: Mechanical characteristics of FLAMs using wood byproducts from different sources

Physical and mechanical properties	FLAMs*			
	CC 1:8 (Casted)	CW1	CW2	CC 1:8 (3D-printed)
Bulk Density g/cm^3	0.37±0.02	0.31±0.02	0.41±0.03	0.40±0.03
Tensile strength (MPa)	6.12±0.37	1.63±0.04	2.14±0.36	11.31±0.57
Young's modulus (MPa)	263.36±21.56	96.66±22.93	127±52.21	244.10±23.92
Elongation at break (%)	2.34±0.32	1.75±0.39	1.86±0.78	4.63±0.41
Flexural strength (MPa)	17.60±3.50	1.92±0.07	2.84±0.50	15.03±0.56
Flexural modulus (GPa)	1.26±0.03	0.13±0.01	0.20±0.01	0.91±0.06
Flexural strain (%)	2.41±0.58	1.96±0.15	1.87±0.38	2.79±0.35
Compressive strength (MPa)	12.09±0.02	1.05±0.01	1.11±0.01	15.31±1.13
Compression Load (kN)	21.77±1.25	2.13±0.13	2.26±0.13	24.43±1.41
Compressive strain (%)	71.38±4.13	10.54±0.63	10.21±0.61	72.99±3.87

* CC 1:8: FLAM prepared by chitosan and cellulose in 1:8 w/w ratio; CW1-FLAM prepared by chitosan and commercial wood flour (W1) in 1:8w/w ratio; CW2-FLAM prepared by chitosan and waste wood flour (W2) in 1:8w/w ratio.

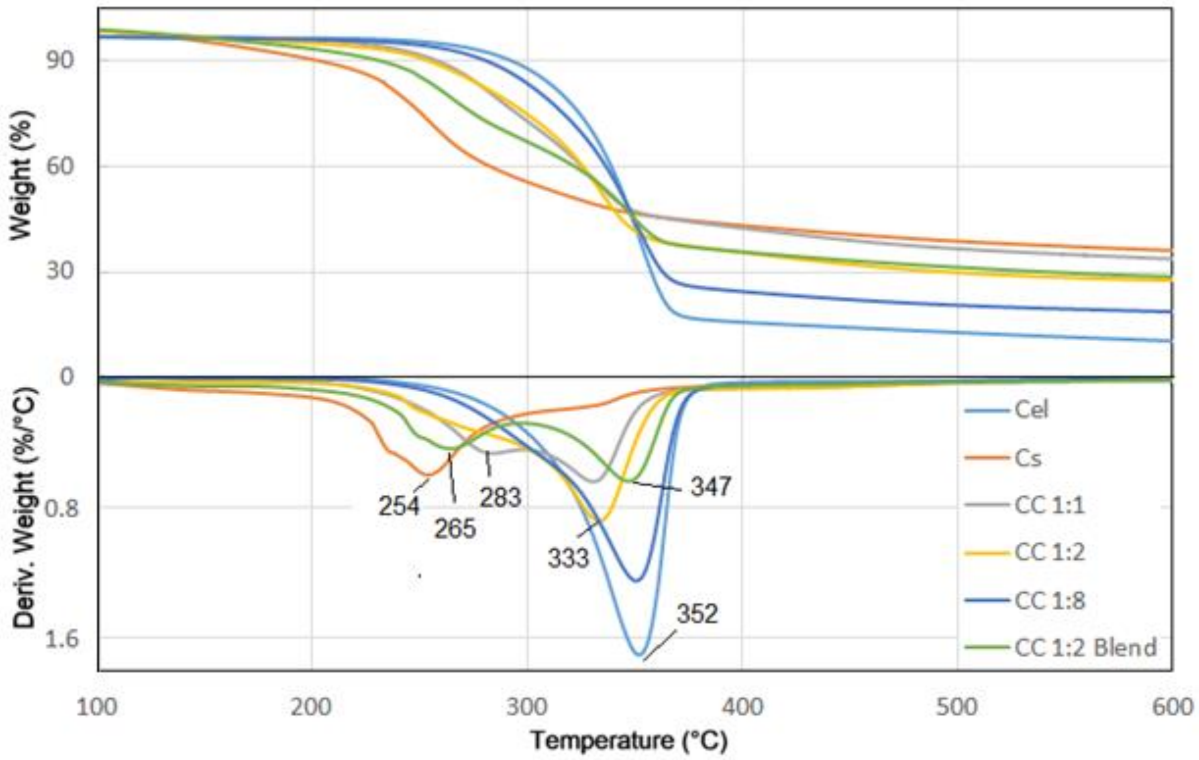

Supplementary Figure 1 | Thermogravimetric analysis (TGA) and derivative thermogravimetric (DTG) curves of chitosan/cellulose composite with different ratio. Physical blend of CC 1:2 showed two distinct mass loss in DTG at 265°C & 347°C (Green line) whereas, FLAM (CC 1:2) showed single mass loss at 333°C (Yellow line). The existence of the single mass loss temperature can be considered as the miscibility indicating interaction of two polymer phases at molecular level after relatively lower concentration of the chitosan (<30%).

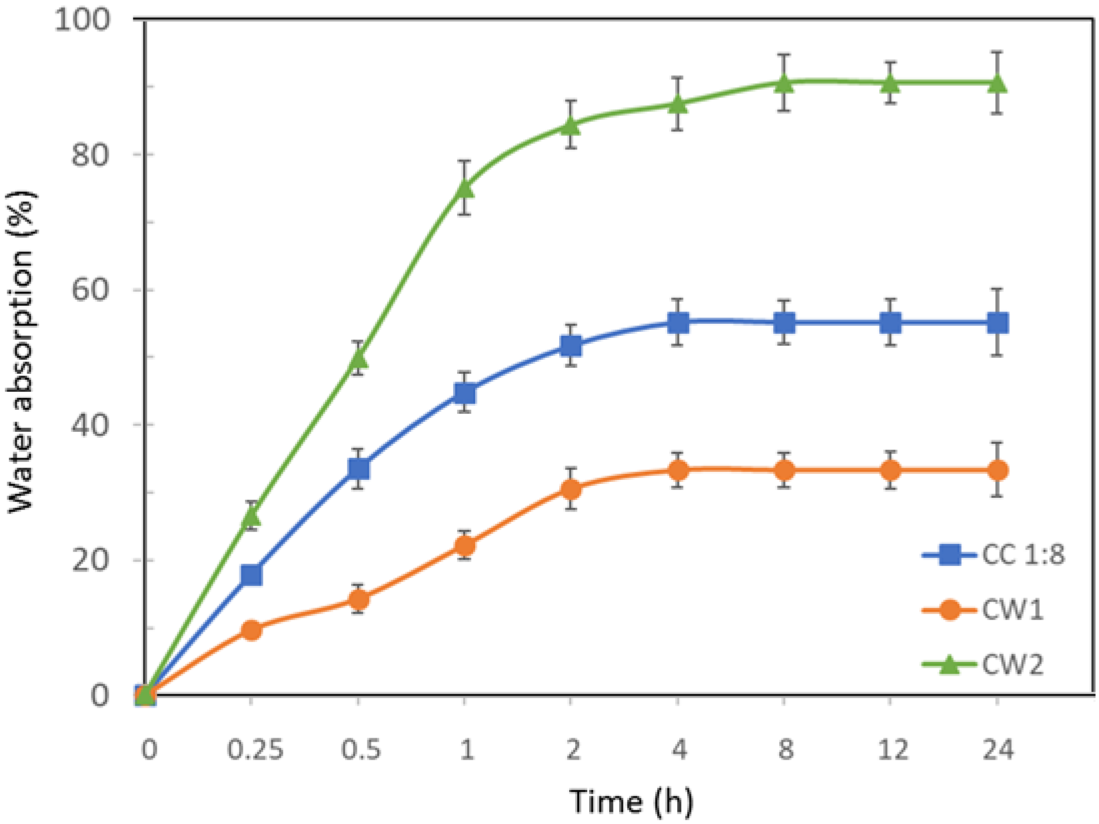

Supplementary Figure 2 | Moisture absorption study of FLAMs. Moisture absorption increased with humidity exposure time until 2h after which it generally showed saturation behavior. The maximum moisture uptake after 24h of exposure were 55.17, 33.33 and 90.62% for CC1:8. CW1 and CW2, respectively.

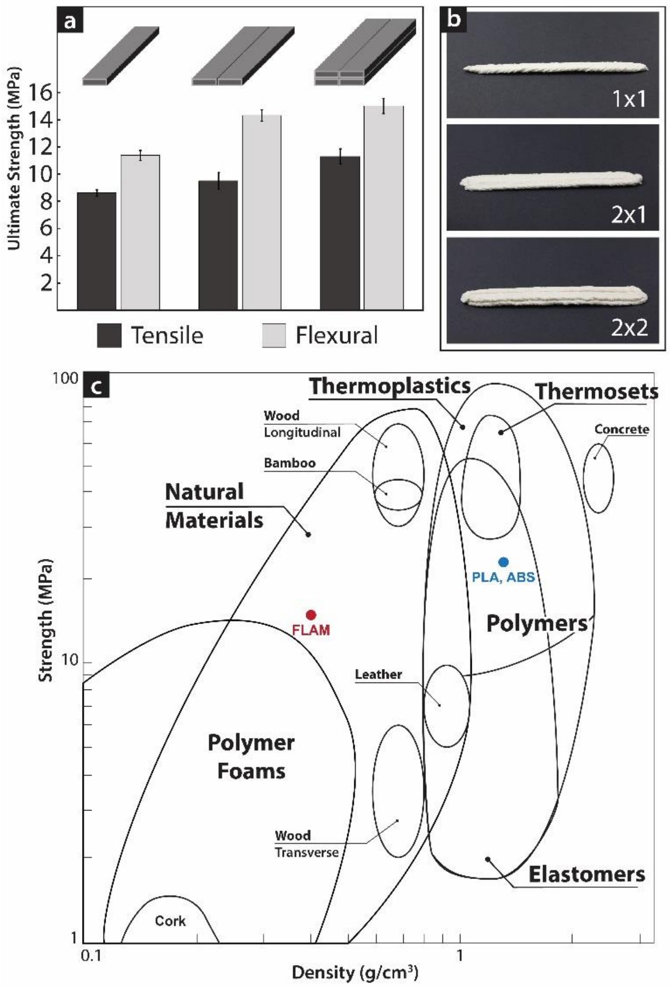

Supplementary Figure 3 | Mechanical characterization of 3D printed FLAM. **a**, Ultimate Tensile and Flexural strengths for 3D printed bars of FLAM. Test specimens were produced by sequential deposition of the material using the system in Supplementary Figure 6. Specimens made of one 3D-printed layer, 2 horizonal layers, and 2 horizontal and two vertical layers, were tested, producing similar mechanical results (Supplementary Movie 1). **b**, Examples of specimens used for the tensile and flexural tests. **c**, Ashby plot of tensile strength Vs. density of common manufacture materials. 3D printed FLAM objects have similar properties than cellulosic natural composites, with an ultimate tensile strength in between the ranges of the longitudinal and transversal values for wood.

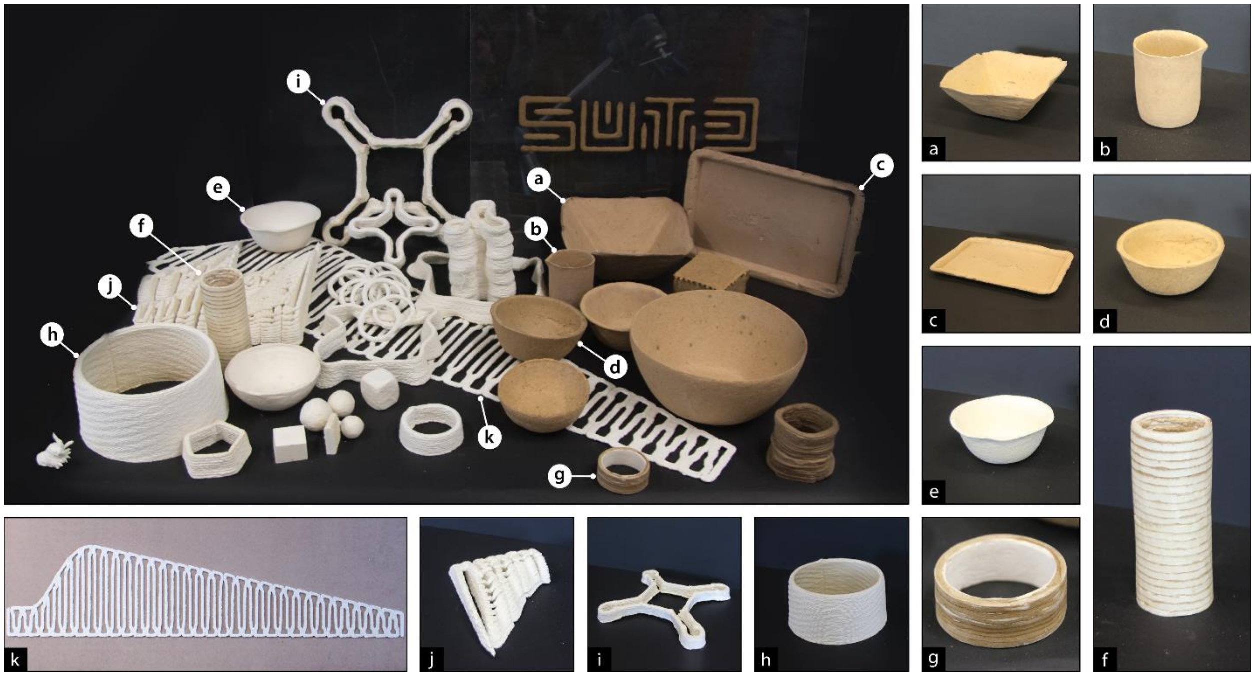

Supplementary Figure 4 | Development prototypes created using conventional, additive manufacturing and combinations thereof methods: (a-d). Household container castings using wood flour FLAM (e) Bowl casting using pure cellulose FLAM, (g-h) Combinations of cellulose and wood-flour, 3D printed by layer, fused and hand finishing. (h) Continuous 3D printing motion and layer compaction evaluation prototype. (i) Autonomous aerial vehicle 3D printed frame. (j) Wind turbine blade core with serpentine motion path and serrated fused edge strategy. (k) Bottom layer of wind turbine blade using serpentine motion path and improved edge fusion strategy.

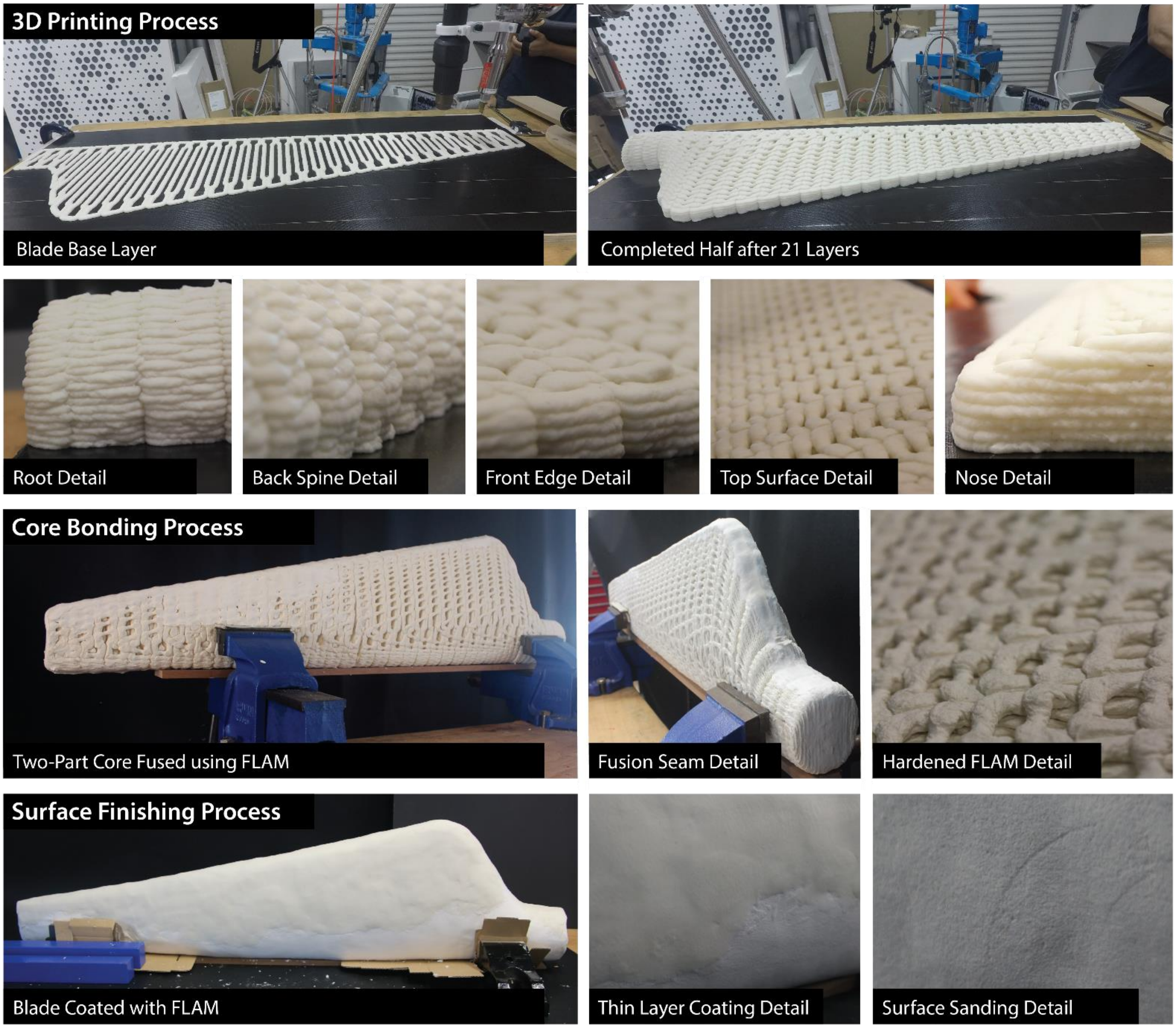

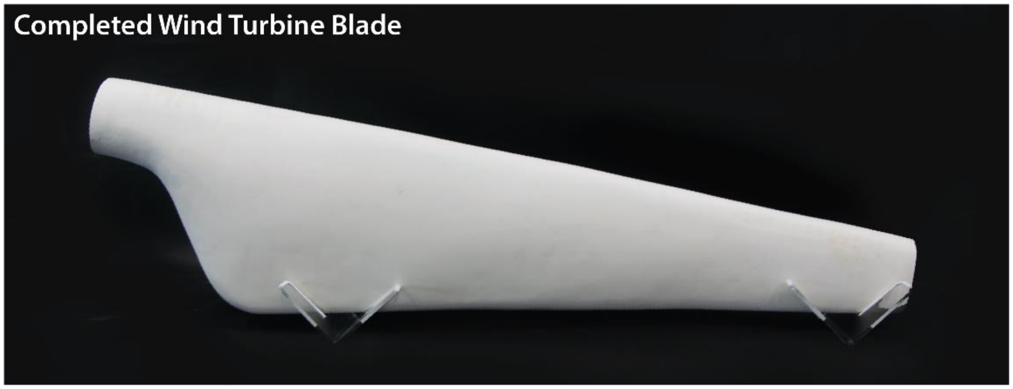

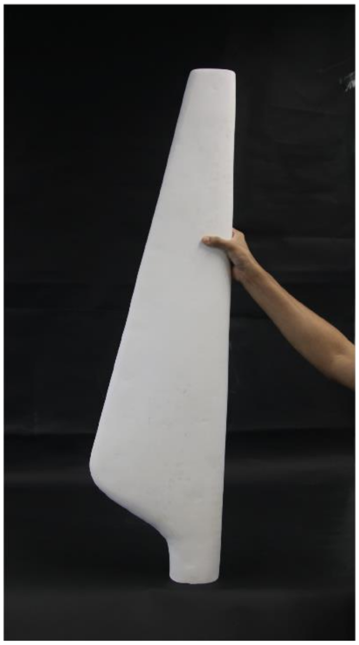

Supplementary Figure 5 | Sequence of turbine blade production combining digital and analog fabrication techniques to demonstrate the versatility of FLAM materials. The core and overall shape were produced by additive manufacturing while bonding, coating and finishing performed using hand tools. The end-product is a lightweight blade 1.2m x 0.3m x 0.1m / 5.28kg entirely made of FLAM.

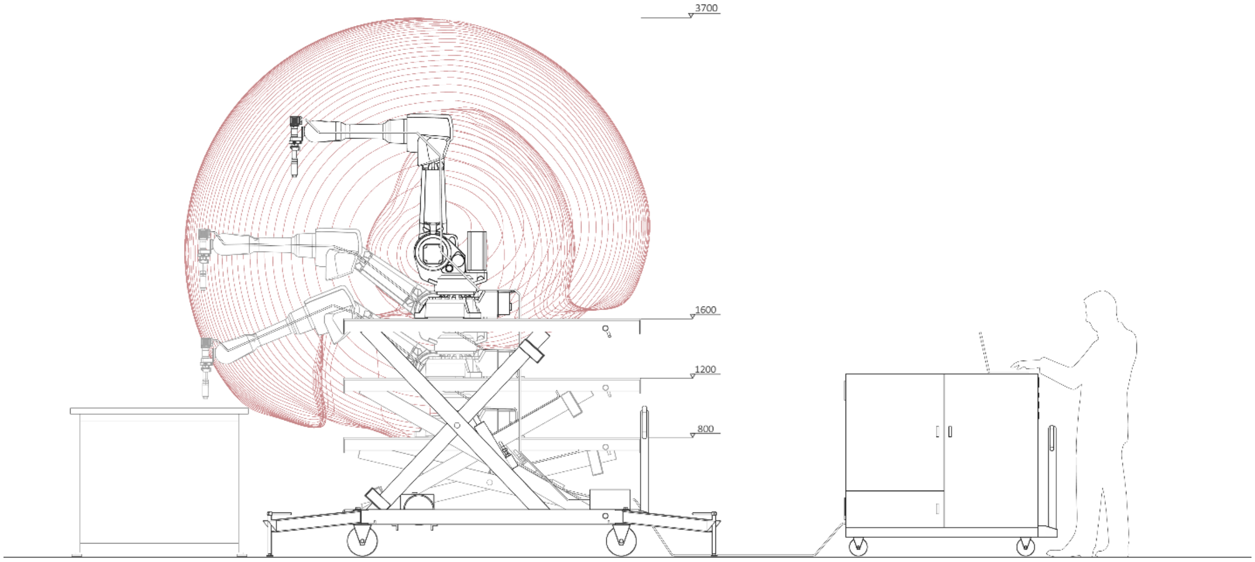

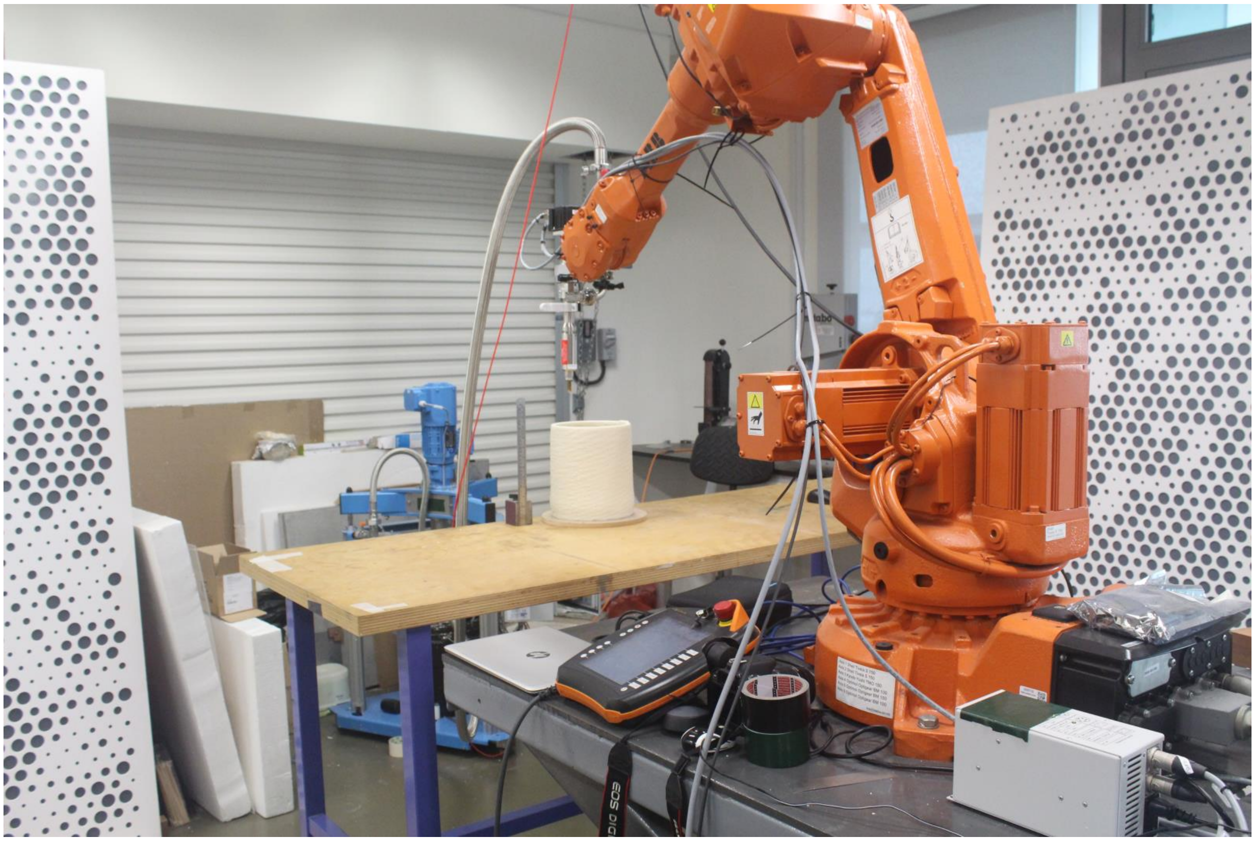

Supplementary Figure 6 | Above: Diagram of the FLAM additive manufacturing setup including kinematic characterization of robot's work envelope at the flange at maximum platform height. Below: Photograph of the system 3D printing a cylindrical shape, including the six-axis robot in orange, mounted on top of the scissor lift mobile platform in gray, and bulk unloading pump in the background in light blue, supplying FLAM through hose to stainless steel dispenser mounted on the robot's flange.

Supplementary Movie 1

Mechanical testing of casted and 3D printed objects. 3-points fracture test of a casted piece of FLAM (t=0s) and a 3D printed specimen (1m26s). Compression tests for a 5×5×5cm 3D printed and sanded down cube of FLAM (2m50s), and a 10cm diameter 3D printed tube (4m01s). Results are summarized on Table S2.

Supplementary Movie 2

Examples of woodworking techniques on FLAM. Sawing of a 40x40x40mm FLAM cube in two halves (t=0s). Drilling of a 0.5cm hole in one of the 40×40×20mm FLAM half cube (54s). Sanding of the preceding half-cube with a 240 grit sanding band (1m38s). Repetition of the same experiment with a block of pinewood of similar characteristics, as comparison with FLAM (2m13s). Hammering of a 6D nail through the other 40×40×20mm FLAM half cube (2m29s).

Supplementary Movie 3

Example of 3D printing speed with FLAM material. A cylinder of 200mm diameter is printed by consecutive deposition of FLAM, reaching a height of 150mm in 10 minutes.

Supplementary Movie 4

Fabrication of a FLAM turbine blade by additive manufacturing. The process starts with the design and path generation using our algorithm specifically developed for FLAM materials (t=0s). The central core of the blade is printed as two separate halves (24s) which are attached together also using FLAM (1m33s). The whole construction is coated with a layer of FLAM (1m52s) and sanded down to a polished finish (2m08s).
